# Genotype-specific reference interval of haptoglobin tests in a Chinese population on the BN II System

**DOI:** 10.1038/s41598-022-20496-x

**Published:** 2023-01-11

**Authors:** Daxin Lei, Saicong Hu, Miao Guo, Jia Wang, Xiaowei Ma, Fukun Wang, Zhengxin He

**Affiliations:** 1 Clinical Laboratory, The 980Th Hospital of PLA Joint Logistical Support Force (Bethune International Peace Hospital), 398 Zhongshan Road, Shijiazhuang, Hebei 050082 People’s Republic of China; 2 Department of Neurology, Hebei Medical University First Affiliated Hospital, Shijiazhuang, People’s Republic of China; 3 Basic Medical Laboratory, The 980Th Hospital of PLA Joint Logistical Support Force (Bethune International Peace Hospital), 398 Zhongshan Road, Shijiazhuang, Hebei 050082 People’s Republic of China

**Keywords:** Medical research, Risk factors

## Abstract

The distribution of Haptoglobin (HP) subtypes differs according to race and geography. It was also confirmed that the serum HP concentration was substantially affected by the HP subtypes. This study aimed to investigate the HP subtypes in northern Chinese and to establish reference intervals for the major HP subtypes using the BN II system. 1195 individuals were included in the study, grouped by haptoglobin subtype, and tested for concentrations by BN II System. Analysis of reference range was performed according to the EP28-A3c guideline. The need to establish reference ranges for subtype, gender, and age groupings was confirmed by the Z-test. The 2.5th and 97.5th percentiles were used as the upper and lower limits of the reference interval, respectively. In the population we investigated, the HP2-2 subtype had the highest proportion, accounting for 49.3%, followed by HP2-1 (38.0%), HP1-1 (7.2%). In addition, about 5.5% of individuals had *HP*^*del*^-related subtypes. The concentrations of the major subtypes (HP1-1, HP2-1, HP2-2) were significantly different, and it was necessary to establish reference ranges by grouping according to the results of the Z-test. The reference intervals were as follows: HP1-1, 0.37–2.19 g/L; HP2-1, 0.38–2.12 g/L; HP2-2, 0.12–1.51 g/L. Significant differences in HP concentrations between genders and ages were found, however, it was not necessary to establish separate reference interval since the results of the Z-test was negative. We have established reference ranges of serum haptoglobin concentrations based on subtypes, which are necessary for the clinical application of haptoglobin.

## Introduction

Haptoglobin (HP) is an abundant acidic glycoprotein in human blood, which is primarily synthesized by hepatocytes. Like the ABO blood group, human HP is a protein with genetic polymorphisms. A typical HP molecule consists of an α Chain and a β Chain connected by disulfide bonds. There are various subtypes of HP proteins due to the genetic variation of the α Chain. Three major subtypes, HP1-1, HP2-1 and HP2-2 are the product of two codominant HP alleles (*HP*^*1*^ and *HP*^*2*^)^1,2^. Casually, a mutation of gene deletion could occur, generating a rare allelotype of *HP*^*del*^ which lacks an approximately 28-kb segment of the haptoglobin-related gene. Thus, *HP*^*del*^ together with *HP*^*1*^ and *HP*^*2*^ give rise to three other HP subtypes, *HP*^*1*^/*HP*^*del*^, *HP*^*2*^/*HP*^*del*^, *HP*^*del*^/*HP*^*del*^^3^. HP subtypes show geographical distribution and population specificity. The HP1 average frequency varies from about 0.56 in Africa to 0.17 in South Asia, with a maximum frequency of 0.87 in Nigeria and a minimum frequency of 0.065 in Calcutta. In Africa, up to 47% of people tested have the *HP*^*del*^ allele. In studies from other regions, it is generally less than 10%^4^. Although there are many studies on HP subtyping around the world, studies based on Chinese populations are rarely reported and the number of participants is small.

HP is an acute-phase reactive protein similar to CRP, which can be used to indicate the stress state of the body, such as myocardial infarction, inflammation, trauma and infection^5^. Another primary function of HP is to bind with free hemoglobin (HB), which confers protection against heme-induced oxidative damage. Any decrease in HP concentration or HB binding capacity may lead to increased renal damage and other diseases^6^. There are differences in HP serum concentrations among normal individuals, which are related to the subtype of HP. Among the three main subtypes, the serum HP concentration of HP1-1 subtype is the highest, followed by HP2-1 and HP1-1^1^. Further, HP subtype also affects HB binding capacity^1^. Thus, differences in HP subtypes provide the possibility, which has been confirmed by many studies, that specific subtype populations are susceptible to particular diseases. For example, People with the HP1-1 subtype appear to be more prone to infections and liver disease, while those with the HP2-2 subtype have a higher risk of cardiovascular disease^2,4^.

The Siemens BN II System is a widely used nephelometric analyzer that offers a broad range of protein assays to evaluate numerous disease states. HP is one of the detection indicators. However, the commercial HP detection kit does not provide a subtype-based reference range, which may reduce the clinical utility of HP. In the present study, we have two primary aims. First, we investigate the distribution of HP subtypes in a northern Chinese population. Second, we formulate the reference range of serum concentrations for the three major HP subtypes.

## Materials and methods

### Participants and study design

**Figure 1:**
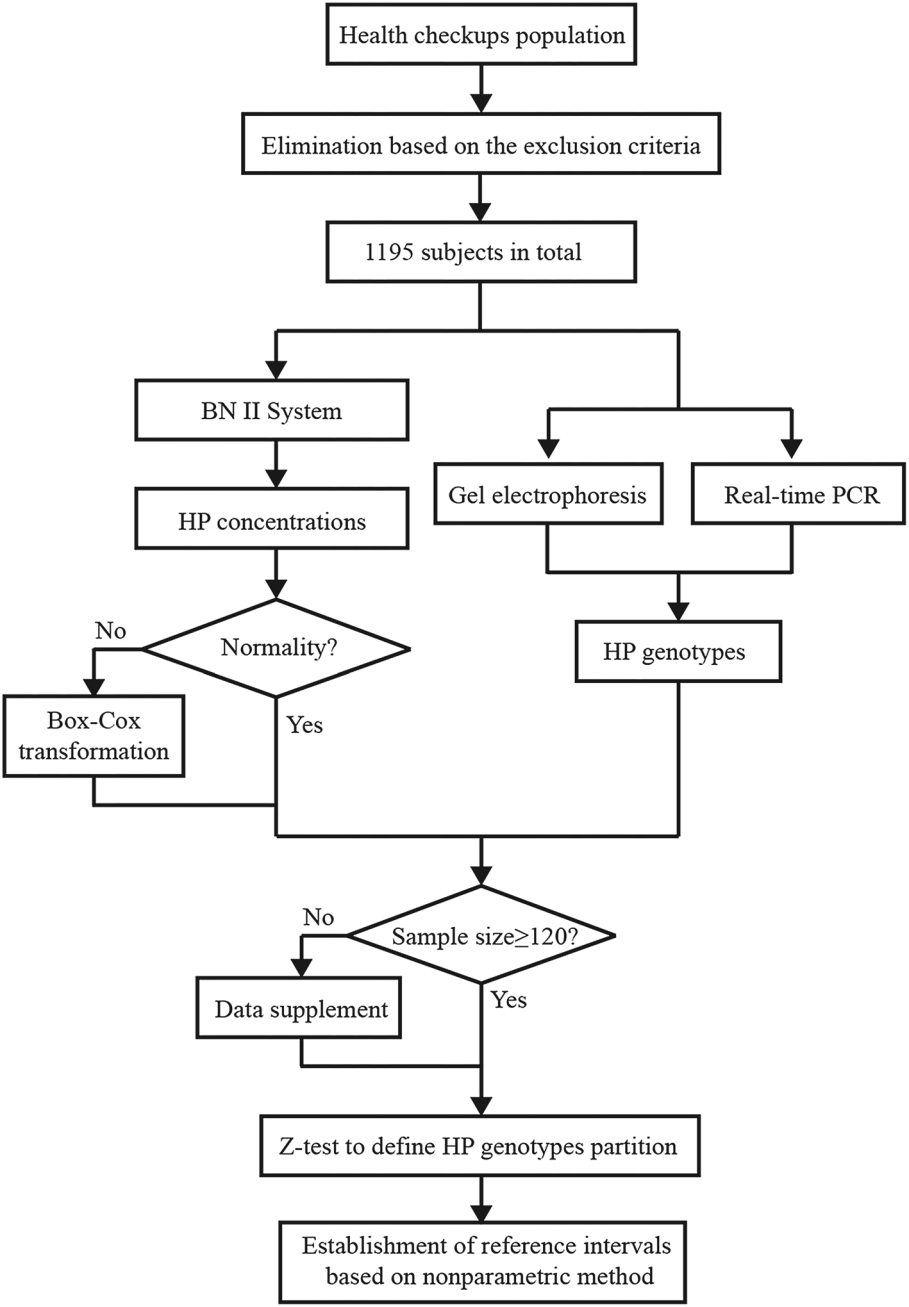
Protocol for establishing HP reference intervals.

This study was designed to investigate the reference intervals of serum HP on the BN II System for different subtype individuals. Totally 1195 individuals who underwent health checkups in the Bethune International Peace Hospital, a local hospital with annual outpatients exceeding 1 million, were enrolled in this study. Before blood collection, they were instructed to maintain a normal diet within 3 days and fast overnight (> 8 h). The exclusion criteria were set as follows: white blood cell (WBC) > 9.5 G/L, HB < 90 g/L, alanine transaminase (ALT) > 50 U/L, cholesterol (CHO) ≥ 5.69 mmol/L, Triglyceride (TG) ≥ 2.26 mmol/L, low-density lipoprotein (LDL) ≥ 4.14 mmol/L, glucose (GLU) > 6.1 mmol/L. The values of the inspection indicators in the exclusion criteria refer to the standards of the National Health Commission of the People’s Republic of China^7–10^. Individuals with any test value meeting the above criteria were excluded from this study. We then determined the HP subtype and serum concentration of each sample and established the reference range based on the Clinical and Laboratory Standards Institute (CLSI) EP28-A3c guideline^11^. The protocol of this study is summarized in Fig. 1. The Institutional Ethics Review Board of Bethune International Peace Hospital granted ethical approval in accordance with the Declaration of Helsinki and waived the need for informed consent since it was a retrospective study.

### Clinical chemistry and HP concentration measurement

Serum HP concentrations were measured by BN II System (Siemens AG, Munich, Germany). The lower detection limit of HP concentration is 0.08 g/L. WBC, HB levels were determined by XN-2000 (Sysmex, Kobe, Japan). ALT, CHO, TG, LDL, GLU measurements were determined by AU 5800 (Beckman Coulter, Brea, USA). Measurement and quality control reagents were matching products. All instruments underwent regular maintenance, calibration, quality control according to ISO9001 and manufacturers’ instructions.

### HP genotyping

The HP subtypes were determined by gel electrophoresis method and real-time PCR method described in a previous study^12,13^.

The serum sample (10 μL) was mixed with 2 μL of 10% HB solution and stood for 5 min at room temperature for forming the HP-HB complexes. The mixture was equilibrated with 12 μL of buffer composed of 125 mM Tris-Base (pH = 6.8), 20% (wt/vol) glycerol, and 0.001% (wt/vol) bromophenol blue and then run on a native polyacrylamide gel. The stacking gel was 4% polyacrylamide in 125 mM Tris-Base (pH = 6.8) and the separating gel was 4.7% polyacrylamide gel in 360 mM Tris-Base (pH = 8.8). Electrophoresis was performed at an initial voltage of 120 V, and the voltage was adjusted to 150 V when the bromophenol blue front reached the separating gel. Gels were stained in freshly prepared solutions containing 5 mL of 0.2% (wt/vol) 3,3′,5,5′-tetramethylbenzidine in methanol, 0.5 mL dimethyl sulfoxide, 10 mL of 5% (vol/vol) glacial acetic acid, 1 mL of 1% (wt/vol) potassium ferricyanide and 150 μL of 30% (wt/wt) hydrogen peroxide^12^.

The results of the gel method and the HP deletion subtype (*HP*^*1*^/*HP*^*del*^, *HP*^*2*^/*HP*^*del*^, *HP*^*del*^/*HP*^*del*^) were confirmed by the TaqMan-based real-time PCR method. The temperature profile was 95 °C for 10 min, followed by 40 cycles of 95 °C for 10 s and 60 °C for 1 min. The sequences of the primers and probes are those described in previous studies^13,14^.

### Statistical analysis

Statistical analysis was performed using R Studio (R Studio, Version 4.1.2, Boston, USA) and SPSS (Version 21). Reference values were determined according to CLSI EP28-A3c guidelines^11^. Outlying observations were removed using the Dixon-Reed method. Because the distribution of HP concentrations in our study was non-Gaussian, determined by the Kolmogorov–Smirnov test, 2.5th and 97.5th percentiles were accepted as the lower and upper limits according to the nonparametric method with sample size ≥ 120. Harris & Boyd’s Z-test (only two groups) and weighted alternative Z-test^15^ (more than two groups) were used to decide whether to partition reference intervals by subclass (sample size per group ≥ 120). Before the Z-test, the subclass data had been Box-Cox transformed using the car (companion to applied regression) package in R if the distributions were non-Gaussian. In addition, If the ratio between the SD in each subclass exceeded 1.5, partitioning of reference intervals was recommended. The 90% confidence intervals (CI) were given by the observed values corresponding to certain rank numbers for the nonparametric method^16^. Since the nonparametric method is used to determine the reference range, the data with a concentration of 0.08 g/L (actually should be less than or equal to 0.08 g/L) will not affect the analysis if the lower limit of the reference value is greater than 0.08. Data were presented as mean ± standard deviation (Mean ± SD). T tests and Analysis of Variance (ANOVA) were used for comparison of two and over two groups of data, respectively.

## Results

### Basic characteristic of the individuals

We randomly selected 1195 healthy individuals for the analysis of HP subtypes and serum concentrations. The included population ranged in age from 19 to 84 with a mean age of 39. About 45% of the individuals were female (532 in 1195). The examination indexes and corresponding average values of the included population are as follows: WBC, 5.78G/L; HB, 141 g/L; ALT, 16.28U/L; CHO, 4.24 mmol/L; TG, 0.97 mmol/L; LDL, 2.52 mmol/L /L; GLU, 5.09 mmol/L.

### Serum HP concentration correlates with subtype

The most common subtype in our study is *HP*^*2*^/*HP*^*2*^ (49.3%), followed by *HP*^*2*^/*HP*^*1*^ (38.0%), *HP*^*1*^/*HP*^*1*^ (7.2%), *HP*^*2*^/*HP*^*del*^ (4.1%) and *HP*^*1*^/*HP*^*del*^ (1.3%). Only two samples were *HP*^*del*^/*HP*^*del*^ genotype. Genotype frequencies in our study were in Hardy–Weinberg equilibrium in all samples (p = 0.80) and also in samples without the deletion allele (p = 0.94).

**Figure 2:**
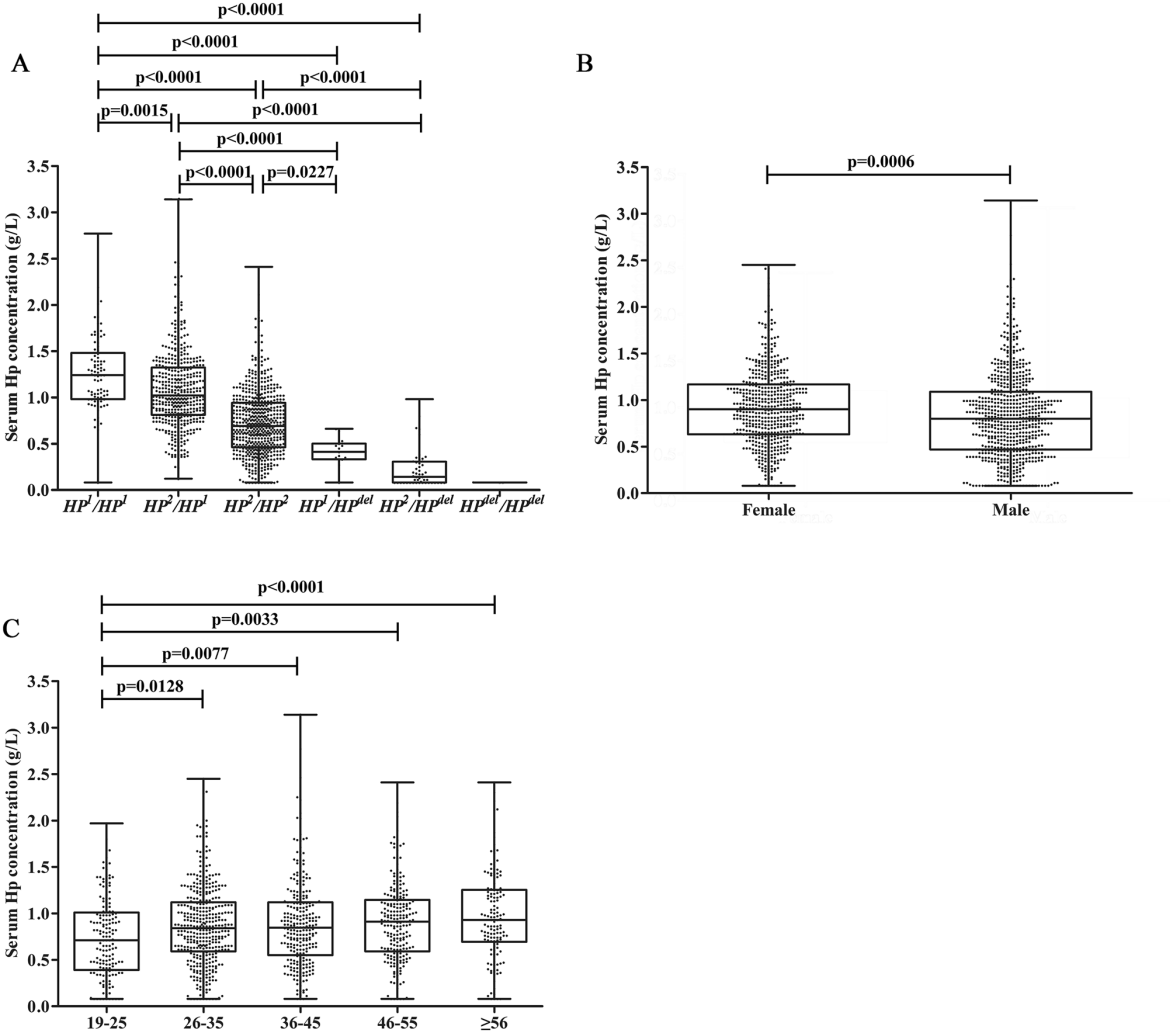
Distribution of HP concentration among various subtypes (**A**), genders (**B**), and ages(**C**). Data are presented by box & whiskers (median + 25 & 75 percentiles, min to max).

Figure 2A indicated that there were differences in the concentration distribution of each genotype. The detailed values are shown in Table 1. It can be found that the *HP*^*del*^ allele or the *HP*^*2*^ allele affects serum HP levels. Genotypes containing the *HP*^*del*^ allele had significantly lower HP serum concentrations (0.26 ± 0.21 g/L) than genotypes without the deletion allele (0.91 ± 0.43 g/L, p < 2.20 × 10^–16^). Moreover, there were significant differences in the concentration of each genotype (Fig. 2A). The *HP*^*del*^/*HP*^*del*^ genotype was not included in the analysis due to the small sample size.

**Table 1:** The relationship between HP subtype and serum concentration.

Genotypes	No. of sample	Percentage (%)	Serum HP concentration (g/L)
**The major subtypes**			
*HP*^*1*^/*HP*^*1*^	86	7.2	1.26 ± 0.40
*HP*^*2*^/*HP*^*1*^	454	38.0	1.09 ± 0.42
*HP*^*2*^/*HP*^*2*^	589	49.3	0.72 ± 0.35
**The subtypes with** ***HP***^***del***^			
*HP*^*1*^/*HP*^*del*^	15	1.3	0.41 ± 0.14
*HP*^*2*^/*HP*^*del*^	49	4.1	0.22 ± 0.20
*HP*^*del*^/*HP*^*del*^	2	0.2	0.08

*p* values were calculated for the three major subtypes (*HP*^*1*^/*HP*^*1*^*, HP*^*2*^/*HP*^*1*^*, HP*^*2*^/*HP*^*2*^) and the subtypes with *HP*^*del*^ (*HP*^*1*^/*HP*^*del*^*, HP*^*2*^/*HP*^*del*^), respectively. The *HP*^*del*^/*HP*^*del*^ subtype was not included in the analysis because of the small sample size.

### Reference intervals for different HP genotypes

We calculated the reference range of HP concentration when subtypes were not distinguished. No outlier was found in the data. Because the data didn’t follow a Gaussian distribution, we use the nonparametric method that doesn’t require the data to be a normal distribution to estimate upper and lower reference limits. The reference range was 0.09–1.87 g/L, which was similar to that given by the regent manual (0–2 g/L). Samples with HP concentrations above the upper limit of the reference value were mainly *HP*^*1*^/*HP*^*1*^ (4 in 29) and *HP*^*2*^/*HP*^*1*^ (22 in 29) subtypes. Samples with HP concentrations below the lower limit of the reference value were mainly *HP*^*del*^ subtype (20 in 31). Similar results appeared when only samples with the three common subtypes of *HP*^*1*^/*HP*^*1*^, *HP*^*2*^/*HP*^*1*^, and *HP*^*2*^/*HP*^*2*^ were included to determine a reference range which was 0.19–1.92 g/L. Samples with HP concentration less than 0.19 g/L were mainly those with *HP*^*2*^/*HP*^*2*^ subtype (25 in 27), while samples with HP concentration higher than 1.92 g/L were mainly those with *HP*^*1*^/*HP*^*1*^ (4 in 28) or *HP*^*2*^/*HP*^*1*^ (21 in 28) subtype.

For further analysis, we included the data of another 34 samples with *HP*^*1*^/*HP*^*1*^ subtype to supplement the sample size to 120^11^. HP subtypes partition was determined by the weighted alternative Z-test with a result that the calculated z-values of all groups exceeded z* (Table 2). Reference intervals for different subtypes of HP are provided in Table 2. Like the difference in the average value, the upper and lower reference limits of the *HP*^*1*^/*HP*^*1*^ (0.37–2.19 g/L), *HP*^*2*^/*HP*^*1*^ (0.38–2.12 g/L), and *HP*^*2*^/*HP*^*2*^ (0.12–1.51 g/L) subtypes decreased sequentially. The 90% confidence intervals for each subtype were calculated and provided as a measure of the uncertainty of the reference interval estimation.

**Table 2:** Reference intervals for different HP subtypes.

Genotypes	No. of sample	z-value^b^	Lower limit	Upper limit	Lower 90% CI	Upper 90% CI
*HP*^*1*^/*HP*^*1*^	120^a^	10.20	0.37	2.19	0.08–0.7	1.87–2.77
*HP*^*2*^/*HP*^*1*^	454	11.65	0.38	2.12	0.36–0.42	1.97–2.30
*HP*^*2*^/*HP*^*2*^	589	17.94	0.12	1.51	0.08–0.18	1.41–1.73

^a^The sample size for the *HP*^*1*^/*HP*^*1*^ subtype was supplemented to 120 to accommodate the requirements of EP28-A3c guideline.

^b^Weighted alternative approach was used for HP subtype grouping, resulting in one z-value per subclass; The value of z* is 6.60.

### Establishing intervals based on gender or age is not essential

According to CLSI EP28-A3c guideline, separate reference intervals for men and women or for different age groups need to be established only when they are clinically useful or are well-grounded physiologically. Regardless of this precondition, we attempted to analyze the necessity of establishing a separate reference range.

In our study, we found differences in the mean values of HP concentrations by gender and by age (Fig. 2B,C). In detail, as Table 3 and Fig. 2B shows, there were significant differences in HP concentrations between males (0.83 ± 0.47 g/L) and females (0.92 ± 0.42 g/L, p < 0.001). But gender group division of HP concentration reference range proved unnecessary by Z-test with a result that the calculated z didn’t exceed z*. In addition, the ratio of the SDs of HP concentration in the male group and female group didn’t exceed 1.5.

**Table 3:** Results of the Z-test for gender and age groupings.

Analyte	No. of sample	Mean	SD	z-value^a^
**Gender**				
Female	532	0.92	0.42	4.12
Male	663	0.83	0.47	
**Age**				
19–25	168	0.74	0.43	4.33
26–35	407	0.87	0.43	0.03
36–45	264	0.89	0.47	0.56
46–55	222	0.90	0.42	1.40
≥ 56	134	0.97	0.48	2.35

^a^Harris & Boyd’s Z-test was used for gender grouping, resulting in one z-value; weighted alternative approach was used for age grouping, resulting in one z-value per group; The value of z* is 6.69.

We divided the samples into 5 groups by age as follows: 19–25, 26–35, 36–45, 46–55, ≥ 56 years old (sample size ≥ 120 in each group). There is a trend that the average value of HP concentration increased with age (Fig. 2C). But the weighted z value of each group calculated by the weighted alternative Z-test was all less than z* as shown in Table 3, which means it is not necessary to determine the reference ranges of the above-mentioned age groups separately. As a supplement, the ratio between the SDs didn’t exceed 1.5.

## Discussion

Our study investigated the distribution of HP subtypes in Hebei, China, and established the HP concentration reference range for the major HP subtypes. The mean values and reference ranges of HP serum levels in our data were slightly different from other studies, possibly due to inherent differences between populations and instrument detection methods^17–19^. Since the BN II system is widely used as a platform in clinical labs to test HP concentration, the reference ranges for different HP subtypes in the Chinese population are not given in the instructions nor established by research before. It is worth noting that the HP concentration measured by immune-related assays may not be completely accurate due to the different immunoreactivity of various subtypes of HP^26^. However, due to the wide application of the BN II system and the lack of a better detection method, it is still necessary to establish a reference range for different subtypes based on this method.

In this study, we choose some more objective exclusion criteria based on the clinical lab tests. Samples of individuals with any indicator, including WBC, HB, ALT, CHO, TG, LDL, GLU, out of the ranges mentioned in the Methods section were excluded. These indicators stand for the possible inflammation, anemia, liver disease, hyperlipidemia, hyperglycemia, which were reported to have an association with the changes in serum HP concentrations^1,4,20^. Using this exclusion method, we eliminated the effects of subjective judgment errors. However, our failure to take into account the past medical history of the population also had certain limitations.

The statement that HP subtype distributions are related to geography and population are validated in our study. We found the *HP*^*1*^ allele frequency is about 0.28, which was close to the results in East Asia and similar to the data of Beijing, China^4,21^. Furthermore, we reconfirmed the interesting link between HP alleles and serum concentration that the higher the amount of *HP*^*del*^ allele and *HP*^*2*^ allele, the lower the concentration^5,17,18^. In addition, the influence of *HP*^*del*^ allele was greater than that of *HP*^*2*^ allele, with a detailed rule showing that *HP*^*1*^/*HP*^*1*^ subtype had the highest HP level, followed by *HP*^*2*^ /*HP*^*1*^, *HP*^*2*^ /*HP*^*2*^, *HP*^*1*^/*HP*^*del*^, *HP*^*2*^ /*HP*^*del*^, and *HP*^*del*^ /*HP*^*del*^. Regardless of HP subtypes, the upper and lower limits of the HP universal reference range for the population in our study (0.09–1.87 g/L) were less than those of the reference range proposed by the IFCC Committee on Plasma Proteins (0.12–2.15 g/L)^22^. This difference may be attributed to that there are more people with HP2-2 subtype in the Chinese population than in the IFCC data^4^.

Our results suggest that establishing HP reference intervals based on subtypes may increase the HP clinical application. For example, among the numerous reports related to multivariate analysis of coronary heart disease (CHD), some studies analyzed the role of HP concentration and the others analyzed the role of HP subtype^20,23^. These reports may have limitations based on the results of our analysis that HP concentration varies by subtype. It is also noteworthy in the situation of HP as an acute-phase protein and any clinical utility based on HP concentrations in future studies. For example, concentrations that are in the normal range for HP1-1 subtypes may have exceeded for HP2-2 subtypes reference range. Moreover, the HP concentration may not increase in individuals with the del mutation. In this case it is difficult for HP to play the role of acute phase protein.

Gender and age were considered as factors affecting HP concentrations as reported before^24,25^. We observed higher HP concentrations in the females and elder individuals in our study. The possible reason was younger people or men who exercise more have higher hemolysis in the body, resulting in more HP consumption^6,24^. Although there were significant differences in concentration in different gender or age groups, we found the establishment of reference ranges by sex or age subclasses was not necessary according to the partitioning method provided by CLSI EP28-A3c guideline. Kasvosve et al. reported a lower HP concentration reference range in males in a healthy black Zimbabwean population, but this may be due to the larger amount of HP2-2 subtype in male participants in their data. They also found no effect of age on reference values based on their analysis of participants aged 20–92 years^18^. However, further research may be required if the trend of HP concentration increasing with age will be reflected in people under the age of 18. Shahabi et al. reported that HP concentrations in people aged 7–13 were only half of those in people over 18 and confirmed the need to establish reference ranges for ages 7–13, 14–17, and 18–56, respectively^24^.

In conclusion, we established reference ranges of HP for the HP1-1, HP2-1 and HP2-2 subtype in Hebei, China and clearly demonstrated the relationship between HP subtypes and concentrations. The non-essentiality of gender and age as a reference range grouping for HP concentrations was also determined. Our results will provide a more reasonable basis when HP is used as a biomarker of disease or a factor for screening high-risk groups of a certain disease.

## Data Availability

The datasets in this study are available from the corresponding author or in the following website, https://doi.org/10.4121/20152226.v1.
